# CRISPR/Cas9-Mediated Phage Resistance Is Not Impeded by the DNA Modifications of Phage T4

**DOI:** 10.1371/journal.pone.0098811

**Published:** 2014-06-02

**Authors:** Stephanie J. Yaung, Kevin M. Esvelt, George M. Church

**Affiliations:** 1 Wyss Institute for Biologically Inspired Engineering, Harvard Medical School, Boston, Massachusetts, United States of America; 2 Department of Genetics, Harvard Medical School, Boston, Massachusetts, United States of America; 3 Program in Medical Engineering & Medical Physics, Harvard-MIT Division of Health Sciences and Technology, Massachusetts Institute of Technology, Cambridge, Massachusetts, United States of America; New England Biolabs, Inc., United States of America

## Abstract

Bacteria rely on two known DNA-level defenses against their bacteriophage predators: restriction-modification and Clustered Regularly Interspaced Short Palindromic Repeats (CRISPR)-CRISPR-associated (Cas) systems. Certain phages have evolved countermeasures that are known to block endonucleases. For example, phage T4 not only adds hydroxymethyl groups to all of its cytosines, but also glucosylates them, a strategy that defeats almost all restriction enzymes. We sought to determine whether these DNA modifications can similarly impede CRISPR-based defenses. In a bioinformatics search, we found naturally occurring CRISPR spacers that potentially target phages known to modify their DNA. Experimentally, we show that the Cas9 nuclease from the Type II CRISPR system of *Streptococcus pyogenes* can overcome a variety of DNA modifications in *Escherichia coli*. The levels of Cas9-mediated phage resistance to bacteriophage T4 and the mutant phage T4 gt, which contains hydroxymethylated but not glucosylated cytosines, were comparable to phages with unmodified cytosines, T7 and the T4-like phage RB49. Our results demonstrate that Cas9 is not impeded by N^6^-methyladenine, 5-methylcytosine, 5-hydroxymethylated cytosine, or glucosylated 5-hydroxymethylated cytosine.

## Introduction

Bacteria utilize an assortment of anti-phage defense mechanisms, including two that act at the nucleic acid level: restriction-modification and Clustered Regularly Interspaced Short Palindromic Repeats (CRISPR)-CRISPR-associated (Cas) systems. Some bacteriophages have developed extensive modifications to their DNA that enable them to evade host restriction endonucleases. For example, phage T4 replaces each cytosine with hydroxymethylated cytosine (hmC), then glucosylates the hydroxymethyl group to form glucosylated hmC (ghmC) [1]. The bound glucose shelters the phage genome from the host's modified cytosine restriction systems, McrA, McrBC, and Mrr, which recognize methylcytosines and hmCs but not ghmCs [2].

CRISPR-Cas systems also function as endonucleases, though unlike restriction enzymes, their recognition sites are programmable by CRISPR RNAs (crRNAs) [3]. As an adaptive immune system, CRISPR-Cas components incorporate fragments of DNA from invading viruses or plasmids into arrays composed of spacers interspersed with repeats on the genome [4], [5]. In Type II CRISPR systems, transcribed arrays are processed into crRNAs that form a complex with the RNA-guided Cas9 nuclease and a trans-activating RNA (tracrRNA) [6]. The crRNA guides the complex to double-stranded DNA “protospacer” sequences that match the sequence of the spacer and are flanked by a “protospacer adjacent motif” (PAM) unique to the CRISPR system [7]. If spacer-protospacer base-pairing is a close match, Cas9 cuts both strands of DNA, often eliminating the plasmid or phage. We sought to determine whether various DNA modifications known to block restriction systems can similarly impede CRISPR-Cas defenses.

## Materials and Methods

### Bioinformatics search

We derived a list of 1749 unique spacers from several sources: 49 *E. coli* strains with CRISPR structures in the CRISPRdb database (http://crispr.u-psud.fr/crispr/, [8]), 72 strains in the ECOR collection [9], 263 strains isolated from humans or animals in various regions of France [10], and 194 Shiga toxin-producing *E. coli* (STEC) strains [11]. CRISPR array sequences were processed in CRISPRfinder (http://crispr.u-psud.fr/Server/, [12]) to extract spacer sequences.

We performed BLASTn searches (http://blast.ncbi.nlm.nih.gov/, [13]) with a word size of seven optimized for short sequences and an E-value of less than 0.1, which corresponded to roughly at least 14 matched nucleotides in the T2/T4/T6 genomes search and at least 17 matched nucleotides in the all T4-like genomes search. We screened hits by first looking for a concentration of exact nucleotide matches at the 5′ end, which would be consistent with a seven-nucleotide “seed” region that does not tolerate mismatches [14]. Outside the seed sequence, at least five mismatches are tolerated [14], though the upper limit of tolerable mismatches has not been characterized in the *E. coli* CRISPR system. We then checked for a properly oriented *E. coli* Type I-E CRISPR PAM such as AAG, ATG, AGG, and GAG in the targeted sequence.

### Bacterial strains and plasmid construction

In addition to wild-type *E. coli* K-12 MG1655 and *E. coli* B, we used methyltransferase-deficient (*dam^−^/dcm^−^*) *E. coli* K-12 (ER2925, New England Biolabs, Ipswich, MA) and restriction-deficient (*mcrA^−^ mcrBC^−^ mrr^−^ hsdR^−^*) *E. coli* K-12 (ER1821, New England Biolabs). *E. coli* were grown at 37°C in LB broth and supplemented with antibiotics as needed at final concentrations of 100 µg/mL spectinomycin, 30 µg/mL chloramphenicol, 300 µg/mL erythromycin, and 100 µg/mL carbenicillin.

Cells expressing SpCas9 were constructed by transforming in DS-SPcas (Addgene plasmid 48645, [15]), which encodes SpCas9 and its cognate tracrRNA on a backbone with a cloDF13 origin of replication and aadA gene. In the dam/dcm methylation studies, we assembled a compatible protospacer plasmid encoding all five of the target sequences with their PAMs; we placed the control, dam1, and dcm1 sequences after a pBR322 origin of replication, and the dam2 and dcm2 sequences after a bla gene. In the T7 infection assays, the spacer was expressed on DS-SPcas such that there was no separate spacer plasmid. In all other experiments, we maintained the designed spacer on a separate plasmid (based on PM-SP!TB, Addgene plasmid 48650, [15]) that expressed one spacer followed by the SpCas9 repeat on a backbone with a p15a origin of replication and cat gene. When a different resistance marker was needed, we switched cat with EryR.

### Bacteriophage strains and propagation

Phage T7 stock was propagated in *E. coli* K-12 MG1655 and RB49 stock (obtained from H. M. Krisch) propagated in *E. coli* B. Wild-type T4 stock was propagated in *E. coli* K-12 MG1655. Phage T4 gt (a gift from New England Biolabs) is T4 *α-gt57 β-gt14*, which does not have functional α- and β-glucosyltransferases [16]. Because the *E. coli* restriction system recognizes and cleaves hmC, preventing T4 gt from plaquing efficiently, we conducted all experiments involving this phage in the restriction-deficient *E. coli* K-12 host ER1821.

In phage stock preparation, an overnight bacterial host culture was diluted 1∶100 in LB, inoculated with phage, and grown for 2.5–5 hours (during which the turbidity of cultures rose and then fell due to lysis). The lysates were spun down at 8000 x g for 5 minutes at 4°C to remove cell debris. The supernatant was filtered through a 0.45 µM membrane and stored at 4°C.

### Transformation assays

We prepared protospacer and spacer plasmids from a dam*^+^*/dcm*^+^* strain, NEB Turbo (New England Biolabs), and performed transformation assays using *E. coli* K-12 MG1655 bacteria containing the protospacer plasmid and DS-SPcas. After transforming equimolar amounts of each spacer plasmid and selecting for all three plasmids (DS-SPcas, protospacer, and spacer), we quantified the number of transformants relative to a transformed spacer plasmid that did not target the protospacer plasmid. We also reversed the transformation order for one set of experiments; that is, we transformed the protospacer plasmid into *E. coli* already carrying DS-SPcas and each spacer plasmid. We observed comparable numbers of transformants regardless of order. We repeated the same transformations in methyltransferase-deficient *E. coli* K-12 using equimolar unmethylated protospacer and spacer plasmids, which were prepared from *E. coli* K-12 *dam^−^/dcm^−^*. Again, for one set of experiments, we reversed the transformation order and noted similar numbers of transformants.

### Plaque assays and efficiency-of-plating calculations

To characterize the level of phage resistance conferred by Cas9, we infected normalized densities of protected *E. coli* with equal titers of phages and counted the number of plaques. Equal cell densities were obtained by diluting an overnight culture and normalizing to an OD_600nm_ of 0.3 after several hours of growth. We added 2 µL of phage to 120 µL of cells, mixed them thoroughly in 1 mL of 0.6% top agar with appropriate antibiotics within 20 minutes, and poured the mixture onto 3 mL of 1.5% solid agar. Independent experiments were performed with different phage dilutions. To calculate an efficiency of plating (EOP), we divided the phage titer from plating the phage on a protected strain by the phage titer from plating the phage on a susceptible wild-type strain.

## Results

### Natural spacers target phages with modified DNA

We began by attempting to discover naturally acquired spacers in bacteria that target phages known to contain modified DNA. Only a handful of phage families have been identified with completely modified DNA, including *Bacillus subtilis* phage PBS2, *Synechococcus elongates* phage S2L, and *Escherichia coli* phage T4 [17]. Since CRISPR-Cas systems and phages of *E. coli* have been better studied than those of the other bacterial hosts, we focused on 1749 unique *E. coli* spacers in available array sequences from the ECOR collection, Shiga toxin-producing *E. coli* (STEC), and other databases.

**Figure 1:**
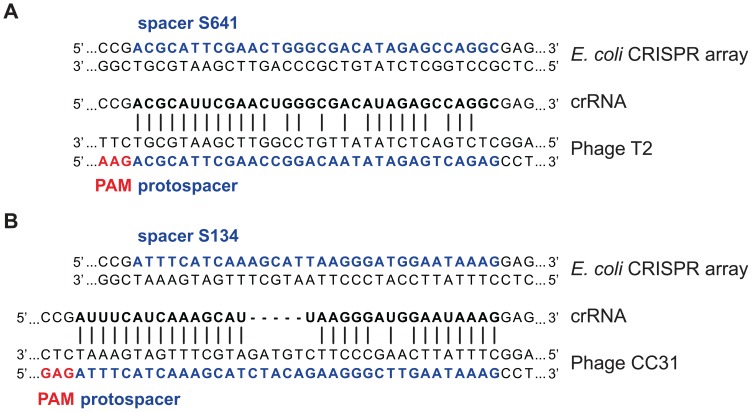
Native *E. coli* spacers target phage with modified DNA. In a BLASTn search, 1749 unique spacers from sequenced *E. coli* CRISPR arrays were queried against T4-like phage genomes. (A) Spacer S641 matches 25 of 32 nucleotides in phage T2. The putative protospacer has a permissible *E. coli* CRISPR PAM AAG and the matching nucleotides are concentrated at the 5′ end as a seed sequence. The spacer originated from the CRISPR1 locus of *E. coli* strain 579, a human-associated isolate from France. (B) Spacer S134 matches 29 of 32 nucleotides in phage CC31. While the protospacer in phage CC31 has five nucleotides inserted in the center of the sequence, there are 15 exactly matched nucleotides at the 5′ end in addition to 14 matched nucleotides after the insertion. The PAM GAG and strongly matched seed region suggest it is a plausible *E. coli* CRISPR target. This spacer was found in several strains, including *E. coli* C str. ATCC 8739, ECOR strains 17 through 21, one farm pig and two human fecal samples in France, duck and cattle fecal samples in Australia [33], and enterotoxigenic *E. coli* (ETEC) strain UMNK88. The spacer and matching protospacer are in blue, the transcribed CRISPR RNA (crRNA) in bold black, and PAM sequence in red.

Upon searching for candidate protospacers in phages T2, T4, and T6, all of which contain ghmC DNA [1], we found one hit that matched 25 of 32 nucleotides in T2′s gene 38, although this spacer was only found in one human-associated *E. coli* (Figure 1A). In an expanded search including T4-like phages, we identified another hit with 29 nucleotides matching phage CC31′s gp35 (Figure 1B). CC31 is the only known non-T-even type phage with predicted glucosyltransferase genes [18], which are required for generating ghmC from hmC. This spacer was found in many different *E. coli* isolates.

The potential presence of natural spacers targeting phage with modified DNA suggests that CRISPR-Cas systems may overcome this form of phage defense. To test this hypothesis, we explored the extent to which the Type II-A *Streptococcus pyogenes* Cas9 (SpCas9), the most commonly used CRISPR-Cas system for genome engineering, is able to cleave various forms of modified DNA.

### Cas9 cuts N^6^-methyladenine and 5-methylcytosine in *E. coli*

**Figure 2:**
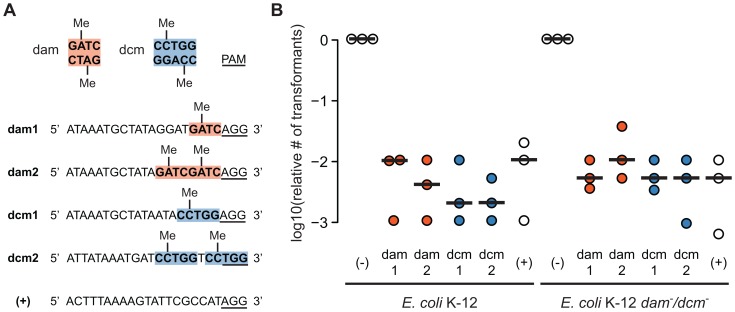
Cas9 cuts methylated cytosines and adenosines in *E. coli*. (A) Synthetic targets were designed to contain one to two dam (orange) or dcm (blue) sites. A control unmethylated sequence (+) was included. The PAM sequence NGG for SpCas9 recognition is underlined. (B) In serial transformations, we selected for the coexistence of DS-SPcas, the protospacer plasmid, and each spacer plasmid. The number of transformants was divided by the number of colonies resulting from a control transformation using a spacer plasmid (-) that did not target the protospacer plasmid. This relative number of transformants is plotted for *E. coli* K-12 and *E. coli* K-12 *dam^−^/dcm^−^* from three independent experiments. Lines represent the median.

DNA adenine methyltransferase (dam) methylates the adenine in 5′-GATC-3′, while DNA cytosine methyltransferase (dcm) methylates the internal cytosine in 5′-CCTGG-3′ and 5′-CCAGG-3′ in *E. coli*. We designed target sequences containing one to two dam or dcm sites as well as a control target sequence with no methylation sites (Figure 2A). We prepared spacer and protospacer plasmids from a dam^+^/dcm^+^ strain and selected for the coexistence of each spacer and its targeted protospacer in transformation assays using dam^+^/dcm^+^ cells expressing SpCas9. All targeted sequences yielded 10^2^ to 10^3^ fewer transformants than the non-targeted control regardless of whether they contained dam or dcm methylation sites (Figure 2B). We observed similar values in methyltransferase-deficient (*dam^−^/dcm^−^*) *E. coli* K-12, in which all plasmids were prepared from a *dam^−^/dcm^−^* strain and were thus unmethylated. Overall, we detected no difference in Cas9 activity on adenine-methylated, cytosine-methylated, and unmethylated target sequences. These results are consistent with reports showing adenine methylation does not affect CRISPR-mediated phage resistance in *Streptococcus thermophilus* [19] and cytosine methylation does not affect SpCas9 activity on sequences with CpG sites in human cells [20].

### Cas9 provides resistance against phages T7 and T4-like RB49 with unmodified DNA

**Figure 3:**
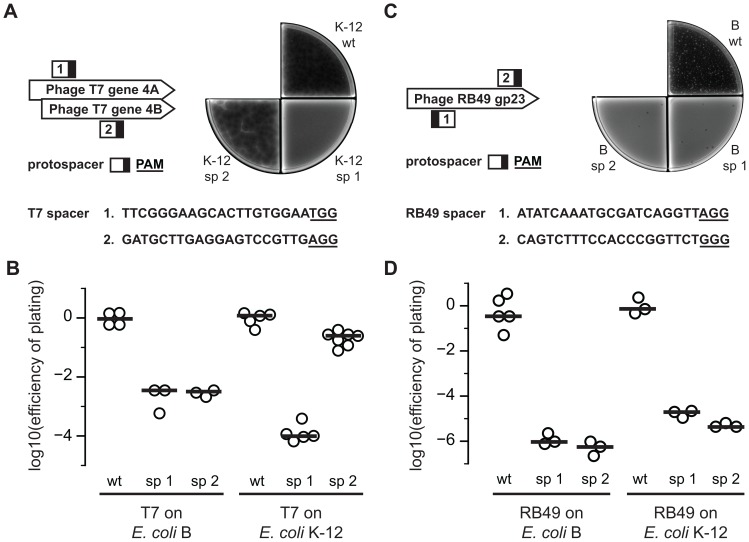
Cas9 reduces *E. coli* susceptibility to phages T7 and RB49. (A) Spacers against T7 were targeted against the primase/helicase gene (gene 4A and 4B). The PAM is underlined in the sequence and shown as a black box in the diagram showing the orientation and location of the protospacer (white box) on the gene. In a representative T7 plaque assay of protected and unprotected strains, there is substantial lysis on wild-type (wt) *E. coli* K-12, visible plaquing on cells with spacer 2 (sp 2), and no plaques on cells with spacer 1 (sp 1). (B) The efficiency of plating of T7 was calculated for each protected strain relative to the unprotected wild-type strain. Independent replicates of *E. coli* B (n = 4, 3, 3) and *E. coli* K-12 (n = 5, 5, 7) are plotted. Lines represent the median. (C) Spacers against RB49 were constructed against the major capsid protein (gp23). In a typical RB49 plaque assay, there is notable lysis on wild-type *E. coli* B, some plaques on cells with spacer 1, and a few plaques on cells protected with spacer 2. (D) The efficiency of plating of RB49 was quantified for each protected strain relative to the unprotected wild-type strain. Shown are independent replicates of *E. coli* B (n = 5, 3, 3) and *E. coli* K-12 (n = 3, 3, 3). Lines represent the median.

We next tested the ability of SpCas9 to provide resistance to lytic phages without DNA modifications by constructing spacers against phages T7 and RB49, neither of which contains modified DNA. RB49 is a T4-like phage that is missing hydroxymethylase and β-glucosyltransferase, which are required for modifying cytosine to hmC and hmC to β-ghmC, respectively [21]. We designed four spacers: two targeting the gene encoding the primase/helicase enzyme of T7 (Figure 3A) and two targeting the gene encoding the major capsid protein of RB49 (gp23), which is one of the most conserved regions across T-even phages [21] (Figure 3C). We transformed each spacer-encoding plasmid into SpCas9-expressing *E. coli* K-12 MG1655 and *E. coli* B to create strains protected from T7 and RB49 infection. We challenged these strains with phage to calculate an efficiency of plating (EOP) compared to unprotected strains; representative plaque plates are included (Figures 3A and 3C).

In *E. coli* B, T7 had an EOP of 10^−3^ on cells expressing spacer 1 or 2 relative to cells without spacers (Figure 3B). In *E. coli* K-12, spacer 1 reduced sensitivity to T7 infection by four orders of magnitude, though spacer 2 only lowered sensitivity by one order of magnitude for unknown reasons. RB49 had an EOP of 10^−6^ on *E. coli* B with spacer 1 or 2, and an EOP of 10^−5^ on *E. coli* K-12 with spacer 1 or 2 (Figure 3D). The decreased plaquing efficiencies of T7 and RB49 on protected strains reflect Cas9 activity against invading unmodified phage DNA.

### Cas9 provides resistance against mutant phage T4 with hmC DNA and wild-type T4 with ghmC DNA

**Figure 4:**
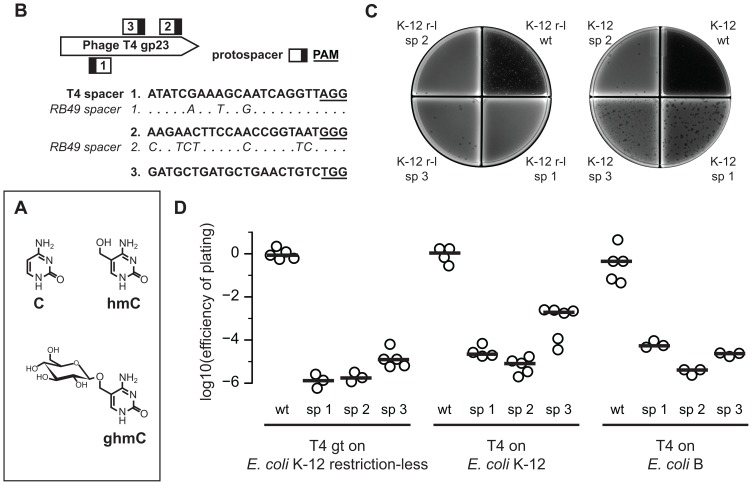
Cas9 reduces *E. coli* susceptibility to phages T4 and T4 gt. (A) The structures of cytosine and modified cytosines are shown. T4 gt has 100% hydroxymethylated cytosines (hmCs). T4 has 100% glucosyl-hydroxymethylated cytosines (ghmCs), specifically 70% α- and 30% β-ghmCs. The ghmC structure shown is in the β-configuration. (B) Spacers against T4 were also designed against the major capsid protein (gp23), which is homologous to that of RB49. For comparison, the RB49 protospacers are aligned below in italics, where dots indicate identical nucleotides. In the T4 sequences, the PAM is underlined. The PAM (black box) and protospacer (white box) are represented on the gene. (C) In a typical plaque assay with T4 gt (left plate), there was complete lysis on wild-type (wt) restriction-less (r-l) *E. coli* K-12 and few plaques on cells with spacers 1, 2, or 3 (sp 1, sp 2, or sp 3). In an assay with T4 (right plate), there was complete lysis on wild-type *E. coli* K-12 MG1655, numerous plaques on cells with spacer 1 or 3, and about a dozen on spacer 2. (D) The efficiency of plating of T4 and T4 gt was quantified for each protected strain relative to the unprotected wild-type strain. Independent replicates of restriction-less *E. coli* K-12 (n = 5, 3, 3, 5), *E. coli* K-12 (n = 4, 4, 5, 6), and *E. coli* B (n = 5, 3, 3, 3) are plotted. Lines represent the median.

Having established that Cas9 can confer resistance against non-modified phage, we proceeded to challenge it with T4 phage containing either hmC or ghmC DNA. During replication, wild-type T4 synthesizes hmC, which contains a hydroxymethyl group attached to the C5 position of cytosine, by using hydroxymethylated dCTP serially converted from dCTP [22]. Then phage-encoded glucosyltransferases add a glucose group to the hydroxymethyl group in α- or β-configuration [23] (Figure 4A). To investigate Cas9 activity against T4 without glucosylated DNA, we included mutant phage “T4 gt”, which has hmC rather than ghmC due to non-functional glucosyltransferases [16]. By using restriction enzymes with varying sensitivity to modified cytosines (according to REBASE, http://rebase.neb.com/), we confirmed that our stocks of phage T4 had ghmC, phage T4 gt had hmC, and phage RB49 did not have ghmC or hmC (Figure S1).

Since T4 gp23 is homologous to gp23 from RB49, we modified our two spacers against RB49 to match the sequences of T4, and also designed an additional spacer (Figure 4B). We tested these spacers using efficiency-of-plating experiments as before; representative plaque plates are shown (Figure 4C). Assays involving T4 gt used restriction-less *E. coli* K-12 because wild-type K-12 restricts hmC DNA; the EOP of T4 gt on *E. coli* K-12 MG1655 is 10^−4^ compared to T4 on MG1655 (Figure S2). In restriction-less *E. coli* K-12, T4 gt exhibited an EOP of 10^−6^ to 10^−5^ on cells carrying any one of the three spacers (Figure 4D). Wild-type T4 displayed an EOP of 10^−5^ on *E. coli* K-12 MG1655 with spacers 1 or 2, and an EOP of 10^−3^ on cells expressing spacer 3. On *E. coli* B with any three spacers, T4 had an EOP of 10^−6^ to 10^−4^. As the difference in EOP values for both T4 gt and wild-type T4 phages were comparable to those of the non-modified T4-like phage RB49, our results demonstrate that SpCas9 is not impeded by hydroxymethylation or glucosyl-hydroxymethylation of phage DNA.

## Discussion

Our discovery that *S. pyogenes* Cas9 is insensitive to methylation, hydroxymethylation, and glucosyl-hydroxymethylation renders it unique among current genome-targeting technologies, as both zinc-fingers (ZFs) and transcription activator-like (TAL) effectors can be engineered to discriminate 5-methylcytosine from cytosine [24], [25]. This difference may be useful for biotechnological applications.

In our bioinformatics search for candidate natural spacers, we were only able to identify two possible sequences against T4-like phages. This type of bioinformatics search is hampered by the currently limited knowledge of specificity and tolerability of mutations in both the acquisition and interference stages of CRISPR systems. While this paper was under review, Fineran et al. published a report exploring the robustness of the *E. coli* CRISPR system, in which degenerate target regions with up to 13 mutations in the protospacer and PAM can promote “priming,” a positive-feedback mechanism to incorporate new spacers based on mutated or outdated spacers [26]. This suggests more lenient bioinformatics searches would be allowable. Furthermore, our search is limited by available sequences of *E. coli* and phages known to modify their DNA, as well as the possibility that these isolates do not encounter T4-like phages in their environments. Future searches may provide additional evidence of CRISPR-based immunity to DNA-modifying phages.

Interestingly, we observed that different spacers conferred differing levels of resistance against phage infection. Since mutations in the protospacer or PAM can allow phage to escape [27], [28], we sequenced Cas9-targeted regions of plaques that appeared on protected strains. Indeed, T4 and T7 plaques on protected *E. coli* had mutated one nucleotide in the PAM, or one to two nucleotides in the protospacer (Table S1). Less effective spacers may be targeting sequences that are more readily mutated, though we cannot rule out the non-mutually exclusive possibility that Cas9 acts more slowly on certain sequences and thus allows phage-induced lysis to outpace Cas9-enabled protection. In *S. thermophilus* CRISPR1 and CRISPR3 systems, the uncut phage genome can still be observed in bacteriophage-insensitive mutants [29], [30]. Further investigation of how some but not all phage DNA molecules escape Cas9 cutting during phage infection is needed.

While phages may inactivate CRISPR proteins [31] or encode their own CRISPR-Cas systems [32], we have demonstrated that DNA modifications that normally circumvent bacterial restriction systems do not impede Type II CRISPR systems. Our findings may help explain why DNA modifications remain uncommon among bacteriophages characterized to date whereas nearly half of bacteria have CRISPR structures [8].

## Supporting Information

Figure S1 **Restriction digest of phages.** Phage DNA was extracted by using the Qiagen Blood and Tissue Kit on 200 µL of phage stock. 10 or 20 U of each enzyme (1 µL) was added to 5 µL of 10X CutSmart Buffer (NEB) in a 50 µL reaction volume containing approximately 100 ng of phage RB49 or T4 DNA, or 800 ng of T4 gt DNA. The reactions were incubated at 37°C for 4 hours before visualizing on a 1% agarose gel stained with SYBR Gold. As expected, DraI cuts all RB49, T4 gt, and T4; HpaII and NheI are sensitive to methylated cytosines and only cut RB49; and XbaI has 50% activity on hmC and partially cuts T4 gt. Blue text denotes cutting.
(TIF)

Figure S2 **Efficiency of plating of T4 gt on wild-type** ***E. coli*** **K-12.** Calculated relative to either T4 infecting *E. coli* K-12 or T4 gt infecting restriction-less *E. coli* K-12, phage T4 gt forms plaques on *E. coli* K-12 at four orders of magnitude less efficiently (red data points). As a general comparison of restriction-modification versus Cas9-mediated protection, Cas9 provides around an order of magnitude greater resistance to phage infection on average, though the level of resistance varies by sequence (blue data points). Independent replicates (n = 11, 5, 5, 5, 4, 15) are plotted; lines represent the median. Cas9+ data were compiled from experiments with various spacer sequences as described in the main text.
(TIF)

Table S1 **Phage escapee analysis.** We picked 13 plaques that formed on Cas9-protected host *E. coli* strains and sequenced the targeted region to identify any mutations. The PAM sequences are underlined. Mutations are in bold text and double-underlined.
(DOCX)
