# Supplementary figures and images for: CRISPR/Cas9-Mediated Phage Resistance Is Not Impeded by the DNA Modifications of Phage T4
Source: PLoS One. 2014 Jun 2;9(6):e98811. doi: 10.1371/journal.pone.0098811 (PMC4041780; doi:10.1371/journal.pone.0098811)

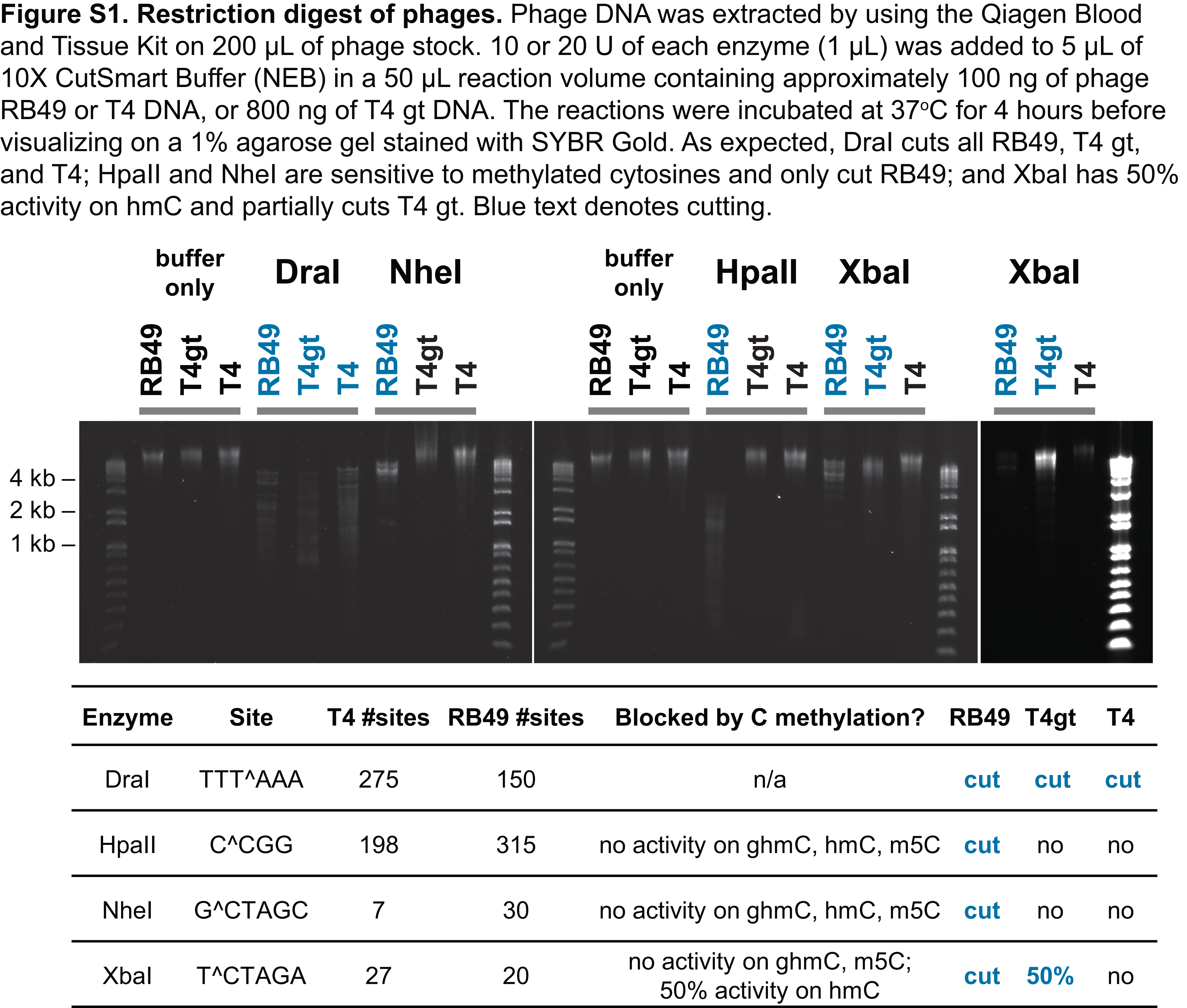

Supplement: Figure S1 — Restriction digest of phages. Phage DNA was extracted by using the Qiagen Blood and Tissue Kit on 200 µL of phage stock. 10 or 20 U of each enzyme (1 µL) was added to 5 µL of 10X CutSmart Buffer (NEB) in a 50 µL reaction volume containing approximately 100 ng of phage RB49 or T4 DNA, or 800 ng of T4 gt DNA. The reactions were incubated at 37°C for 4 hours before visualizing on a 1% agarose gel stained with SYBR Gold. As expected, DraI cuts all RB49, T4 gt, and T4; HpaII and NheI are sensitive to methylated cytosines and only cut RB49; and XbaI has 50% activity on hmC and partially cuts T4 gt. Blue text denotes cutting. (TIF) [file pone.0098811.s001.tif]

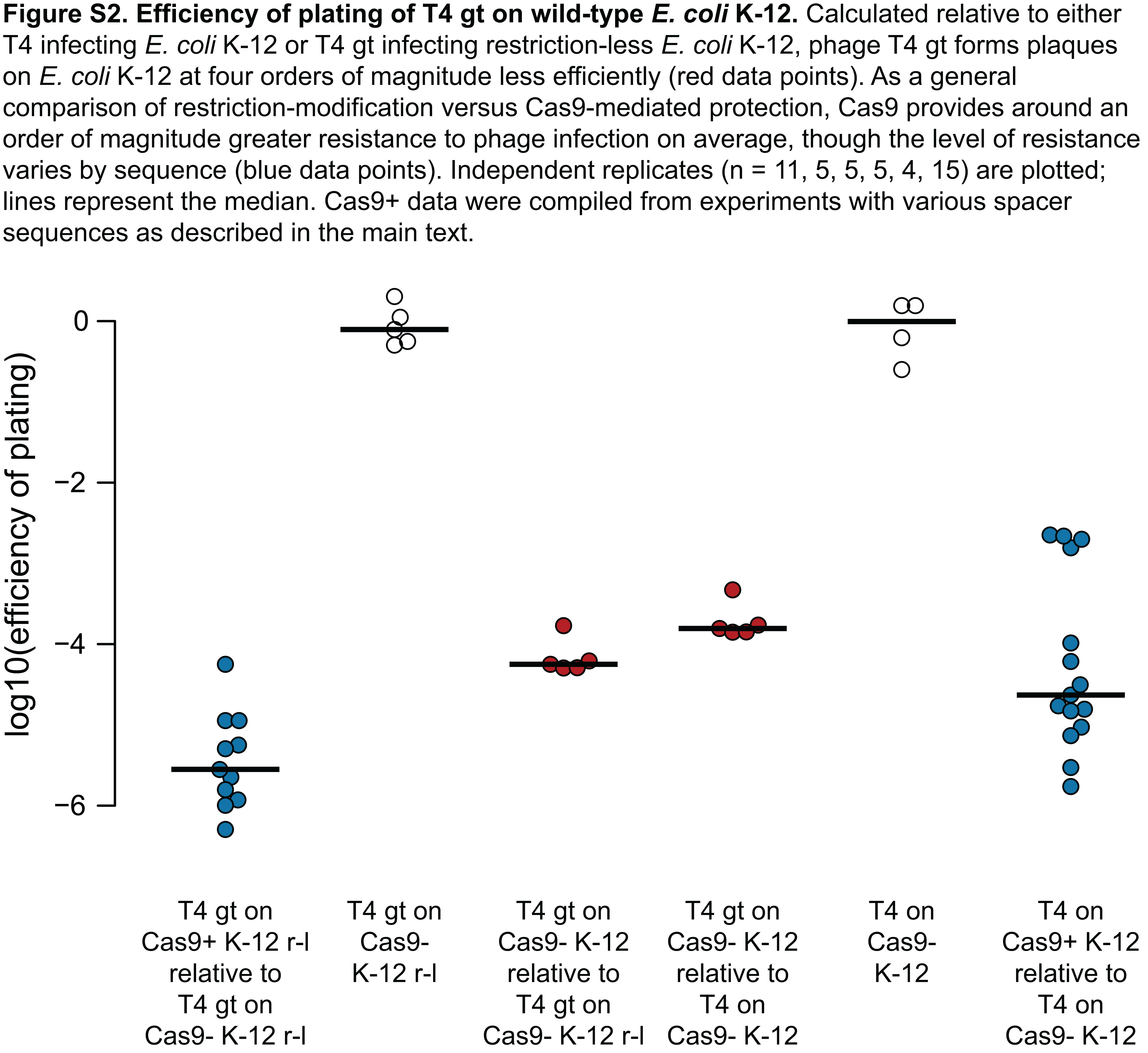

Supplement: Figure S2 — Efficiency of plating of T4 gt on wild-type E. coli K-12. Calculated relative to either T4 infecting E. coli K-12 or T4 gt infecting restriction-less E. coli K-12, phage T4 gt forms plaques on E. coli K-12 at four orders of magnitude less efficiently (red data points). As a general comparison of restriction-modification versus Cas9-mediated protection, Cas9 provides around an order of magnitude greater resistance to phage infection on average, though the level of resistance varies by sequence (blue data points). Independent replicates (n = 11, 5, 5, 5, 4, 15) are plotted; lines represent the median. Cas9+ data were compiled from experiments with various spacer sequences as described in the main text. (TIF) [file pone.0098811.s002.tif]
