# Supplementary material for: CRISPR/Cas9-Mediated Phage Resistance Is Not Impeded by the DNA Modifications of Phage T4
Source: PLoS One. 2014 Jun 2;9(6):e98811. doi: 10.1371/journal.pone.0098811 (PMC4041780; doi:10.1371/journal.pone.0098811)
Supplement: Table S1 — Phage escapee analysis. We picked 13 plaques that formed on Cas9-protected host E. coli strains and sequenced the targeted region to identify any mutations. The PAM sequences are underlined. Mutations are in bold text and double-underlined. (DOCX) [file pone.0098811.s003.docx]

**Table S1. Phage escapee analysis.** We picked 13 plaques that formed on Cas9-protected host *E. coli* strains and sequenced the targeted region to identify any mutations. The PAM sequences are underlined. Mutations are in bold text and double-underlined.

| **Phage** | **Target** | **Spacer-PAM sequence** | **Mutation in phage** | **Host** |
| --- | --- | --- | --- | --- |
| T4 | spacer 1 | ATATCGAAAGCAATCAGGTTAGG | ATATCGAAAGCAATCA**C**GTTAGG | ER1821 |
| T4 gt | spacer 1 | ATATCGAAAGCAATCAGGTTAGG | ATATCGAAAGCAATCAGGTTAG**C** | ER1821 |
| T4 | spacer 2 | AAGAACTTCCAACCGGTAATGGG | AAGAACTTCCAACCGGTAATGG**C** | MG1655 |
| T4 | spacer 2 | AAGAACTTCCAACCGGTAATGGG | AAGAACTTCCAACCGGTAATGG**C** | MG1655 |
| T4 | spacer 3 | GATGCTGATGCTGAACTGTCTGG | GATGCTGATGCTGAACTGTCTG**A** | MG1655 |
| T4 | spacer 3 | GATGCTGATGCTGAACTGTCTGG | GA**A**GCTGATGCTGAACTGTCTGG | MG1655 |
| T4 gt | spacer 3 | GATGCTGATGCTGAACTGTCTGG | GATGCTGATGCTGAACTGTCTG**T** | ER1821 |
| T7 | spacer 1 | TTCGGGAAGCACTTGTGGAATGG | TTCGGGAAGCACTTGTGGAATG**T** | MG1655 |
| T7 | spacer 1 | TTCGGGAAGCACTTGTGGAATGG | TTCGGGAAGCACTTGTGGAAT**T**G | MG1655 |
| T7 | spacer 2 | GATGCTTGAGGAGTCCGTTGAGG | GATGCTTGAGGAG**A**CCG**C**TGAGG | MG1655 |
| T7 | spacer 2 | GATGCTTGAGGAGTCCGTTGAGG | GATGCTTGAGGAG**A**CCG**C**TGAGG | MG1655 |
| T7 | spacer 2 | GATGCTTGAGGAGTCCGTTGAGG | GATGCTTGAGGAG**A**CCG**C**TGAGG | B |
| T7 | spacer 2 | GATGCTTGAGGAGTCCGTTGAGG | GATGCTTGAGGAG**A**CCG**C**TGAGG | B |
|  |  |  |  |  |
